# The complete chloroplast genome sequence of *Populus tremuloides* (Salicaceae)

**DOI:** 10.1080/23802359.2019.1688121

**Published:** 2019-11-08

**Authors:** Ang Li, Zhe Hou

**Affiliations:** Key Laboratory of Southwest China Wildlife Resources Conservation (Ministry of Education), China West Normal University, Nanchong, China

**Keywords:** *P. tremuloides*, chloroplast genome, phylogenetic analysis, genetic information

## Abstract

The complete chloroplast genome sequence of *Populus tremuloides* was characterized from Illumina pair-end sequencing. The chloroplast genome of *P. tremuloides* was 155,816 bp in length, containing a large single-copy region (LSC) of 85,804 bp, a small single-copy region (SSC) of 16,489 bp, and two inverted repeat (IR) regions of 26,962 bp. The overall GC content is 36.71%, while the correponding values of the LSC, SSC, and IR regions are 64.9%, 69.2%, and 60.3%, respectively. The genome contains 167 complete genes, including 86 protein-coding genes (77 protein-coding gene species), 73 tRNA genes (29 tRNA species) and 8 rRNA genes(4 rRNA species). The Neighbour-joining phylogenetic analysis showed that *P. tremuloides* and *Populus tremula* clustered together as sisters to other *Populus* species.

## Introduction

*Populus tremuloides* (Salicaceae) is the most geographically widespread and ecologically important tree species in the Northern Hemisphere, which have largely persisted inanundomesticated stateand and is highly resistant to different environmental stresses (Hou et al. 2018). *Populus tremuloides* distribute widely and can adapt to different climates and environments owing to without anthropogenic influence and harbor a wealth of genetic variation. *Populus tremuloides* habors high ecological and economic value and have high level of intraspecific genetic diversity (Callahan et al. 2013). Therefore, *P. tremuloides* is an excellent system for understanding genetic information and genome varition patterns (Neale and Antoine 2011). Moreover, we can develop conservation strategies easily when we understand the genetic information of *P. tremuloides*. In the present research, we constructed the whole chloroplast genome of *P. tremuloides* and understood many genome varition information about the species, which will provide beneficial help for population genetics studies of *P. tremuloides*.

The fresh leaves of *P. tremuloides* were collected from Ottawa city, Ontario province, Canada (45°25′N, 75°41′W). Fresh leaves were silica-dried and taken to the laboratory until DNA extraction. The voucher specimen (*P. tremuloides*002) was laid in the Herbarium of China West Normal University and the extracted DNA was stored in the −80 °C refrigerator of the Key Laboratory of Southwest China Wildlife Resources Conservation. We extracted total genomic DNA from 25 mg silica-gel-dried leaf using a modified CTAB method (Doyle 1987). The Illumina HiSeq 2000 platform (Illumina,San Diego, CA) was used to perform the genome sequence. We used the software MITObim 1.8 (Hahn et al. 2013) and metaSPAdes (Nurk et al. 2017) to assemble chloroplast genomes. We used *P. tremula* (GenBank: NC_027425) as a reference genome. We annotated the chloroplast genome with the software DOGMA (Wyman et al. 2004), and then corrected the results using Geneious 8.0.2 (Campos et al. 2016) and Sequin 15.50 (http://www.ncbi.nlm.nih.gov/Sequin/).

The complete chloroplast genome of *P. tremuloides* (GenBank accession number MN561844) is 155,816 bp in length, containing a large single-copy region (LSC) of 85,804 bp, a small single-copy region (SSC) of 16,489 bp, and two inverted repeat (IR) regions of 26,962 bp. The overall GC content is 36.71%, while the corresponding values of the LSC, SSC, and IR regions are 64.9%, 69.2%, and 60.3%, respectively. The chloroplast genome contains 167 complete genes, including 86 protein-coding genes (77 protein-coding gene species), 73 tRNA genes (29 tRNA species) and 8 rRNA genes (4 rRNA species). Most of the genes occur as a single copy, except for 35 gene species occur in double copies.

**Figure 1.**
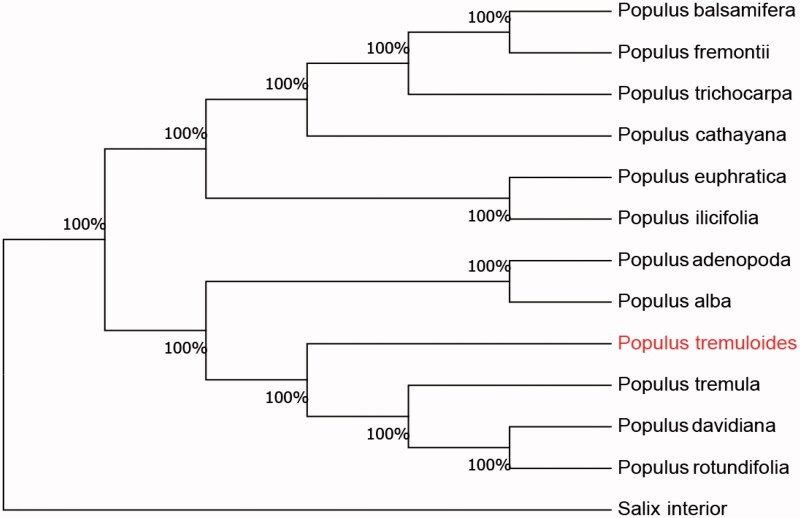
Neighbour-joining (NJ) analysis of *P. tremuloides* and other related species based on the complete chloroplast genome sequence. Genbank accession numbers: *P. tremula* (KP861984), *P. davidiana* (KX306825), *P. yunnanensis* (KP729176), *P. euphratica* (KJ624919), *P. adenopoda* (NC032368), *P. rotundifolia* (KX425853), *P. cathayana* (KP929175), *P. balsamifera* (KJ664927), *P. ilicifolia* (NC031371), *P. trichocarpa* (EF489041), *P. fremontii* (KJ664926), and *Salix interior* (NC024681).

We used the complete chloroplast genomes sequence of *P. tremuloides* and 11 other related species of *Populus* and Salix interior as outgroup to construct phylogenetic tree. 13 chloroplast genome sequences were aligned with MAFFT (Katoh and Standley 2013), and then the Neighbour-joining tree was constructed by MEGA 7.0 (Kumar et al. 2016). The results confirmed that *P. tremuloides* was clustered with *P. tremula* (Figure 1).
